# Supplementary material for: Prediction of Heart Function and Volume Status in End-Stage Kidney Disease Patients through N-Terminal Pro-Brain Natriuretic Peptide
Source: Medicina (Kaunas). 2022 Jul 22;58(8):975. doi: 10.3390/medicina58080975 (PMC9331554; doi:10.3390/medicina58080975)

Supplementary Figure S1: Comparison of ΔN-terminal pro-brain natriuretic peptide (ΔNT-proBNP) concentration of angiotensin II receptor blocker (ARB) and β-blocker use. Values are expressed as median values. BP, blood pressure.

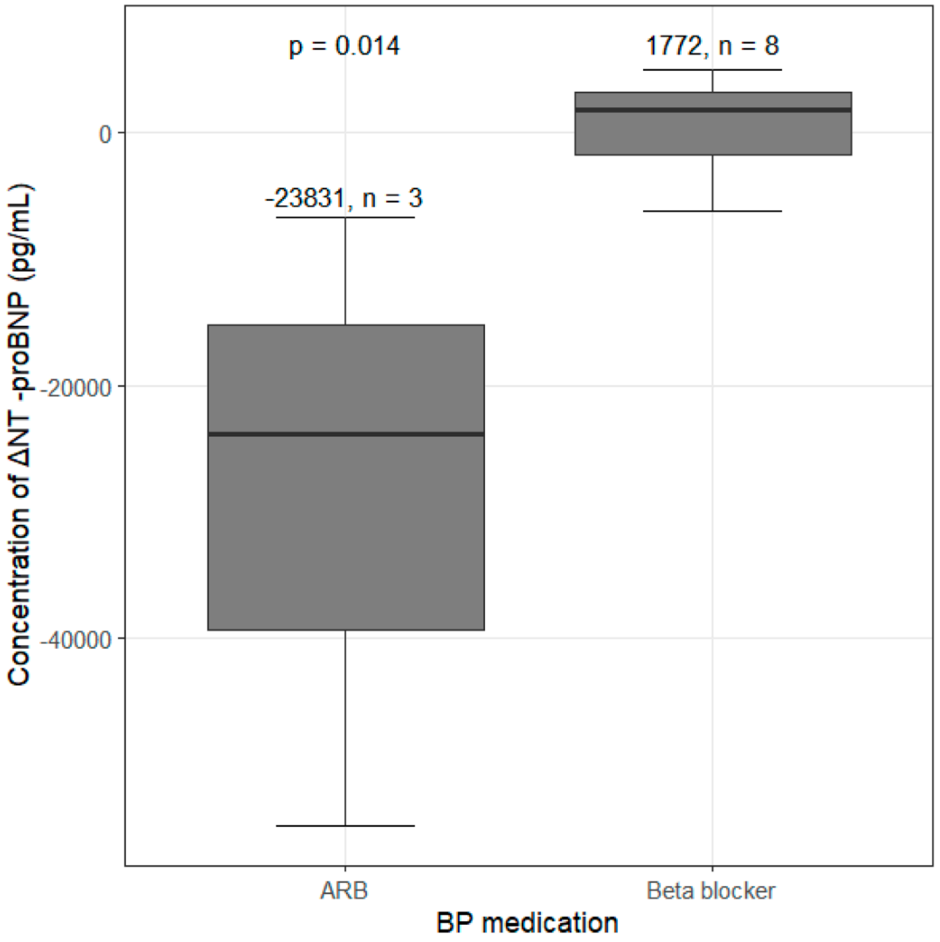

Supplement: Supplementary file 1 [file medicina-58-00975-s001.zip › Supplementary Figure S1.pdf]
