# Supplementary figures and images for: Prediction of Heart Function and Volume Status in End-Stage Kidney Disease Patients through N-Terminal Pro-Brain Natriuretic Peptide
Source: Medicina (Kaunas). 2022 Jul 22;58(8):975. doi: 10.3390/medicina58080975 (PMC9331554; doi:10.3390/medicina58080975)

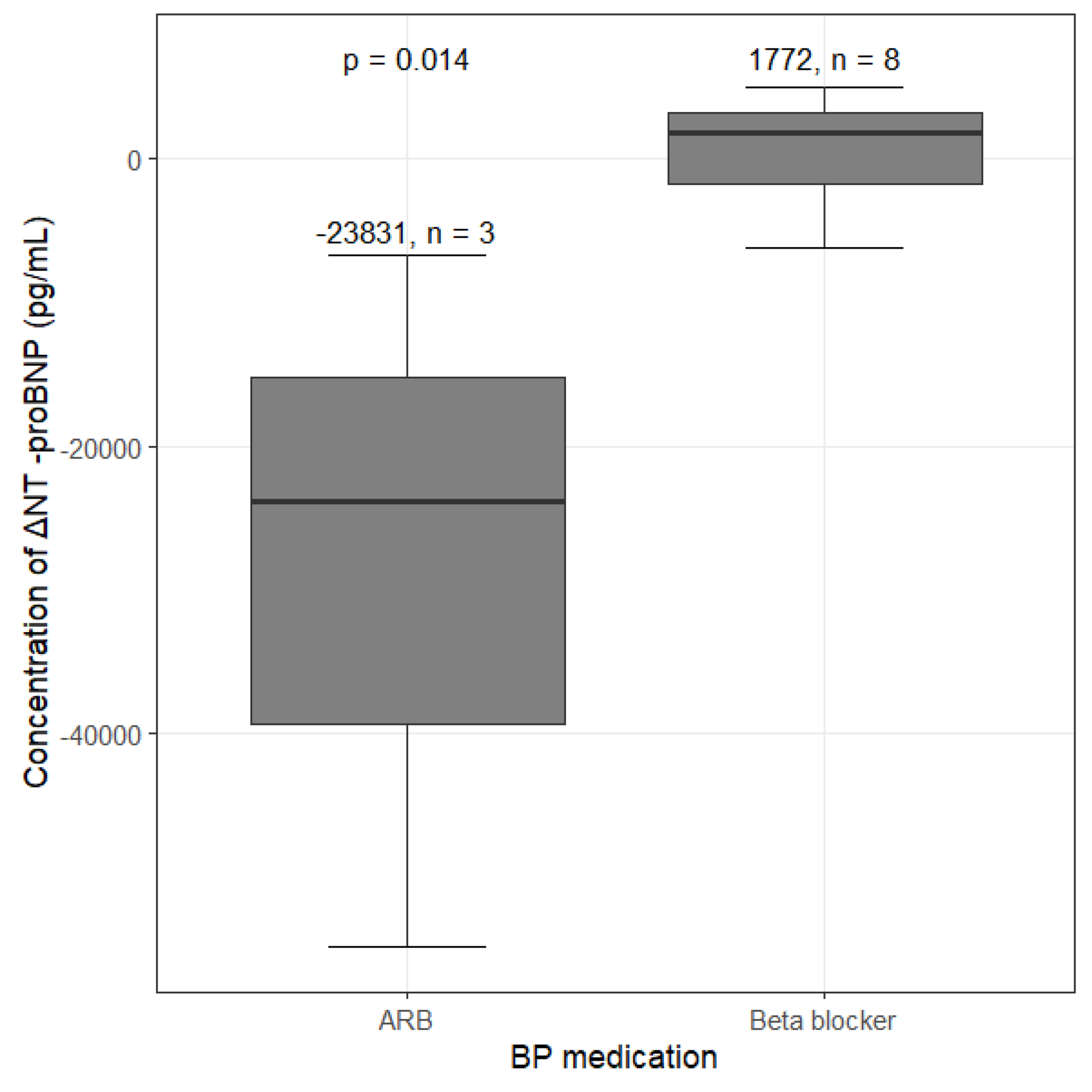

Supplement: Supplementary file 1 [file medicina-58-00975-s001.zip › Supplementary Figure S1.tif]
